# The role and features of peer assessment feedback in college English writing

**DOI:** 10.3389/fpsyg.2022.1070618

**Published:** 2023-06-26

**Authors:** Qiyu Sun, Fang Chen, Shengkai Yin

**Affiliations:** ^1^School of Foreign Languages, Shanghai Jiao Tong University, Shanghai, China; ^2^School of Languages and Linguistics, Faculty of Arts, University of Melbourne, Parkville, VIC, Australia

**Keywords:** peer assessment, feedback take-in, linguistic measures, NLP, college English writing

## Abstract

Peer assessment is a common pedagogical practice for evaluating students’ writing in college English classrooms. However, in-depth research on the learning outcomes after peer assessment is scarce and inconsistent; how peer feedback is used has not been fully explored either. This study compared peer feedback to teacher feedback and explored the different features of feedback and its impact on draft revision. Two specific research questions were answered in this study: (1) In which aspects can peer feedback supplement teacher feedback in improving the linguistic features in writing? (2) What are the differences in features of peer feedback versus teacher feedback? And how do they connect to feedback take-in? Two writing tasks were assigned to 94 students. One received teacher feedback and the other peer feedback. Pre-feedback and post-feedback writings in both tasks (4 sets in total) were scored and human ratings were adjusted using Many-Facet Rasch modeling to control for differences in leniency. Drawing on three natural language processing (NLP) tools, this study also assessed writing qualities by comparing 22 selected indices related to the scoring rubrics for human raters, which involve three dimensions: cohesion, lexical quality and syntactic complexity. Peer and teacher feedback was then coded based on features of feedback to explore their influence on draft revision. The results showed that both peer and teacher feedback had positive effects on rating scores. We confirmed peer feedback as an effective classroom approach to improve writing, though limited compared to teacher feedback as reflected in the indices. In terms of feedback features, students often stopped at identifying the language problems, while the teacher provided more explanations, solutions or suggestions regarding the problems identified. Implications for peer feedback research and implementation of peer assessment activities are provided.

## Introduction

In English classrooms in higher education, peer assessment is a popular practice for evaluating students’ writings because it is hard for the teacher to engage in every student’s writing as frequently as desired, especially when detailed and accurate feedback is essential (Cho and Schunn, 2007; Gielen and De Wever, 2015; Yu and Hu, 2017; Huisman et al., 2018). Peer assessment has also been acknowledged as an approach to delivering feedback to students in an efficient and effective manner (Topping, 1998; van Zundert et al., 2010). A substantial amount of research has been conducted on the role and benefits of peer assessment from various aspects, including its influence on students’ writing self-efficacy (e.g., Fathi et al., 2019; Lee and Evans, 2019), autonomous motivation (e.g., Yousefifard and Fathi, 2021), and writing performance (e.g., Nelson and Schunn, 2009). However, less attention has been paid to the role that peer feedback can or can not play to supplement teacher feedback, let alone an in-depth analysis of feedback features that may account for such possibilities or discrepancies.

Accordingly, this study decides to examine the effects of peer feedback from a different perspective by comparing its role against teacher feedback. It also looks into the feedback features of peer review in the feedback process and compares to those by the classroom teacher for a more valid evaluation of feedback impacts. We hope to illuminate both researchers and teachers on an efficacious deployment of peer assessment in English as a foreign language (EFL) classroom settings, focusing on writing progress.

## Literature review

### Features of peer feedback as a communication process in peer assessment

The term “peer feedback” is commonly used interchangeably in the literature with other terminologies such as peer review, peer response, peer grading, peer evaluation, peer critique, and peer assessment. It is necessary to distinguish it from peer assessment for the current study. According to Liu and Carless (2006), peer feedback refers to a “communication process through which learners enter into dialogues related to performance and standards” (p. 2). Despite this process-centric interpretation, it is also conceptualized as the “information learners can use to improve the quality of their work or learning strategies” (Winstone et al., 2022, p. 224). Peer assessment is defined as “an arrangement for learners to consider and specify the level, value, or quality of a product or performance of other equal-status learners, then learn further by giving elaborated feedback and discussing their judgments with peers to achieve a negotiated agreed outcome” (Topping, 2017, p. 2). Therefore, the distinction between the two terms lies in that peer feedback is about the communication process and all the concrete information produced in this process, such as direct error correction or simply comments, but without a formal score; whereas peer assessment involves both peer feedback and a final grading. In this study, for example, peer assessment is the activity that generated rating scores in addition to feedback in words that justified the ratings. The latter is the focus of this study.

Feedback feature is defined as the “structural components of feedback comments” (Wu and Schunn, 2020, p. 3). It has been widely studied under a variety of classification frameworks where different feedback features were believed to exert different influences on the implementation of feedback in writing instruction, though most of them were conducted in first language setting (Faigley and Witte, 1981; Nelson and Schunn, 2009; Lu and Law, 2012; Gielen and De Wever, 2015; Elizondo-Garcia et al., 2019; Wu and Schunn, 2020), with fewer studies in foreign langauge or second language context (Strijbos et al., 2010). However, scholars in this line of research do not seem to place much emphasis on the distinction between feedback classification in native or non-native context, presumably as a result of the identical function of feedback.

The most fundamental classifications of peer feedback features are binary, including surface-level versus meaning-level (Faigley and Witte, 1981), evaluative versus informational (Narciss, 2008), simple versus elaborated (Narciss, 2008; Strijbos et al., 2010), cognitive versus affective (Nelson and Schunn, 2009), informative versus suggestive (Gielen and De Wever, 2015), and form versus content (Elizondo-Garcia et al., 2019).

More complicated classifications were also available, some with new schemes that were extracted from previous studies, others with more features within the binary classifications. For instance, Hattie and Timperley (2007) did a meta-analysis on feedback effects, based on which they proposed an overarching four-level model, comprising personal evaluation, product evaluation, learning process, and self-regulation. Lu and Law (2012), on the other hand, explained an extended cognitive-affective classification and proposed identification, explanation, suggestion and language for the cognitive category, and critical and positive comments for the affective category.

Several studies are especially relevant to peer feedback for writing, such as Nelson and Schunn (2009), Lu and Law (2012), and Wu and Schunn (2020, 2021). They all explored cognitive feedback which is to our interest and they all included a language component. Nelson and Schunn (2009) and Wu and Schunn (2021) both described five features, one of which was about language. However, Nelson and Schunn named it “affective language” which further breaks into praise and mitigating compliments, while Wu and Schunn (2021) listed a mitigating praise in parallel with four other cognitive types: identification, explanation, general suggestion and specific solution. Language was part of the cognitive features in Lu and Law (2012), which was defined as “comments addressing the writing in general” (p. 265), such as pointing out the informal writing style of the language used. Thus, Lu and Law’s intention for the language component matched the rater’s thinking when reading writing samples, which is in line with our exploration context. However, it is Wu and Schunn’s (2020) framework of coding that formally addressed this perspective. Actually, their coding scheme is so thorough and rich, it is worth more explanation below.

Several terminologies in Wu and Schunn (2020) are straightforward and rather friendly, which guided our research plan to a large extent. For example, they coded feedback from several perspectives, including *type*, *feature* and *scope*. The *type* perspective covered praise, summary, and implementable comments. Praise is defined as “purely evaluative remarks on good features” such as “You did good in paragraph 2.” Summary is defined as “statements of what the writer had done,” such as “The writer wrote a compare-and-contrast essay on pets.” If students are to revise their work based on feedback, these comments would not be helpful thus came the term “unimplementable comments.” In contrast, implementable comments were defined as “revision-oriented comments that could trigger revisions,” such as pointing out a concept that is causing reader confusion. When soliciting feedback in the classroom, implementable comments are the key elements that help make the assessment formative and can contribute to teaching and learning (Strijbos et al., 2010; Winstone et al., 2022). They are especially desirable and worth studying and are the actual targets in our research.

The *feature* perspective in Wu and Schunn (2020) classified feedback in six categories: identification, explanation, suggestion, solution, mitigating praises and hedges. The first four were regarded as cognitive feedback while the latter two as affective feedback (Wu and Schunn, 2020). We think these four cognitive features overlapped with implementable comments since they would also trigger and guide revisions. The feature perspective is the focus of another paper a year later (Wu and Schunn, 2021), where suggestion was rephrased as “general suggestion” and solution as “specific solution”. These feature codes are straightforward in terms of meaning and function, thus were adopted for this study. However, we used the term “cognitive type” in this study and used the word “feature” in a general way. Examples of each cognitive feature are provided under the methodology section.

The *scope* perspective dealt with language components and was divided into high-level versus low-level comments. While high-level comments addressed writing devices such as arguments and organization, low-level comments addressed language control and conventions. So, the language component in the model meant differently from the three research described previously. This perspective is actually important for assessing writing and can align with rubrics that usually accompany writing activities.

Although much research has probed into the features of peer feedback, they rarely incorporated the rubrics employed in peer assessment into the analysis of peer feedback features (Faigley and Witte, 1981; Narciss, 2008; Nelson and Schunn, 2009; Strijbos et al., 2010; Gielen and De Wever, 2015; Elizondo-Garcia et al., 2019). This practice is not helpful in integrating evaluation standards or validating the value of peer feedback in the writing class. For example, although Wu and Schunn (2020) considered the language aspect, they did not make a connection between the comments on language (scope) and the rubrics which were available and used for peer rating. It is reasonable to believe that students will refer to the rating rubrics when providing feedback on the language of the writing samples. Whether they stick to the rubric and whether they rely on some aspects of the rubric more than others may shed light on the thinking underneath the rating behavior, which deserves more attention.

Related to the observation above, coding schemes employed in previous studies usually were not fine-grained enough, such as relying on simple dichotomies. We think more can be done on a single data set to extract maximum information from it like what Wu and Schunn (2020) have done. As a result, previous studies leave open how and why peer feedback leads to different aspects in the revision and how it affects the possibility of students integrating the feedback. Furthermore, attention has to be paid to the comparison of features between peer feedback and teacher feedback which is important if we are interested in the appropriate role of peer feedback in supplementing that of teachers in the classroom.

### Effectiveness of peer feedback take-in

Availability is not enough to turn the feedback into effective take-ins, i.e., incorporation of feedback as can be observed in the revised drafts. How and why they are implemented or not implemented is another issue. A plethora of studies have already examined the effects of peer feedback in comparison with that of teachers on students’ revision quality and writing performance (Fathman and Whalley, 1990; Paulus, 1999; Ruegg, 2015; Cui et al., 2021). However, research on the effectiveness and utilization of peer feedback mainly focused on the implementation rate of feedback (Connor and Asenavage, 1994; Paulus, 1999) or pre–post gains in writing scores (Ruegg, 2015; Cui et al., 2021).

Implementation of feedback is one of the most frequently explored areas by peer feedback researchers. For example, Connor and Asenavage (1994) investigated the impact of peer and teacher feedback on essay revisions of first-year English learners in a United States university. It was found that only 5% of the revisions resulted from peer feedback. Similarly, Paulus (1999) reported that peer feedback accounted for only 14% of revisions while teacher feedback accounted for 34%. However, early studies did not categorize the revisions, thus providing a rather restricted evaluation of feedback utility. Later research began to investigate revisions in more detail. For example, Yang et al. (2006) found that peer feedback brought about a higher percentage of meaning-change revisions while teacher feedback brought about more surface-level revisions. Min (2005, 2006) and Altstaedter (2018) revealed that peer reviewers tended to focus on low-level language problems but ignored high-level ones. However, based on our observation in the classroom, we think peer feedback actually leads to more extensive revision behavior that is beyond the immediate implementation of feedback, making it necessary to measure the development of writing performance from a more comprehensive perspective instead of focusing on implementation rate of available feedback only.

Researchers in the field of peer feedback are rather concerned about the research methodology in measuring improvement. Previous attempts to measure improvement in writing performance have focused on score gains between drafts. For example, Ruegg (2015) calculated gains between pre-treatment and post-treatment writing scores, and reported them regarding organization, vocabulary, content, and holistic quality, respectively. Cui et al. (2021, 2022) also used the score gains between drafts as the indicator of revision quality. All gains in these studies were based on the observed scores. However, improvement in scores only does not reveal much on which specific aspects peer feedback can play a role. More seriously, human raters are found to differ in leniency when granting scores (Styck et al., 2021) and teachers sometimes opted for an inflated higher rating to acknowledge the efforts by students which actually misrepresents student progress (Darling-Hammond and Adamson, 2014). When peer assessment is involved and rating scales are narrow, these human bias factors may be amplified which calls for better analytical methods (Eckes, 2009) and more objective measures (Yoon, 2017).

With the advancements in natural language processing (NLP), a diverse range of objective measures of linguistic features have been developed (Ohlrogge, 2009; Vidakovic and Barker, 2010; Lu, 2011; Crossley and McNamara, 2016; Kyle and Crossley, 2018; Paquot, 2019). Their application in writing literature has also proliferated in recent years (Ansarifar et al., 2018; Casal and Lee, 2019). Such fine-grained measures often reveal much valuable information about writing quality and can serve as additional indices to evaluate changes between drafts. Also, using a variety of indices enables sensitive detection of student progress that may be more accurate and convincing (Yoon, 2017). In this study, we decided to apply NLP indices to supplement human rating. Details of the selected indices are described in the methodology section.

### Purposes and research questions of the present study

Although peer feedback has been studied from a number of perspectives, it remains to be learned how effective they are in changing the writing performance of students. Neither do we know the relationship between feedback features and feedback take-in based on fine-grained measures of writing quality and feedback type and scope. This study was therefore designed to add one concrete example with improved methodology for these purposes. Two research questions will be addressed:

Research question 1: In which aspects can peer feedback supplement teacher feedback in improving the linguistic features in writing?

Research question 2: What are the differences in features of peer feedback versus teacher feedback? And how do they connect to feedback take-in?

## Materials and methods

### Participants and instrument

A total of 94 Chinese-speaking English learners (56 males and 38 females) from three parallel classes and three researchers participated in this study. All learners were first-year students in a top university in China with various majors and were placed in the advanced English class based on an English proficiency placement test upon arrival. They have learned EFL for at least 10 years and were about 17–18 years old typically upon admission. One of the three researchers was the classroom teacher and the other two were teaching assistants.

According to the given curriculum, the genre to practice for these classes was an expository essay. All students responded to two expository writing tasks successively with the same rating rubric. Task 1 was an essay titled *Challenges for First-Year X University Students*, while Task 2 was topic-free, in which students wrote on anything that interested them. We made this decision from an ecological point of view (Siedentop, 2002; Tudor, 2003; Huang et al., 2021). First, this was not lab research, and the classroom assessments served instructional and formative purposes in addition to a tool for data collection. It was efficient to discuss the issues in writing using the same topic for the first task which also helped when the teacher shared examples and demonstrated how to give feedback. To stick to the curriculum goal, the second task was still expository writing. Allowing choice in the topic not only motivated students (Katz and Assor, 2007), but we hoped it could also maximize learning opportunities for all students, including incidental learning of the vocabulary and expression during the peer assessment activity. In our opinion, this is what makes peer assessment a good learning tool. In addition, if different topics were used, the assessment procedure might be more fun and there might be a lower chance of formulaic comments by student raters.

All researchers rated students’ writings for both tasks but part of their ratings were for research purpose only. Students received grades from the classroom teacher for Task 1 and from peers on Task 2 on the first drafts, both of which were accompanied by detailed feedback. The scores were awarded holistically with structure, logic, and language in consideration. A rating scale from 0 to 10 (0 being the lowest and 10 the highest) was adopted for the rubric.

### Data collection procedures

Firstly, after several minilectures on essay writing at the beginning of the semester, students were assigned Task 1 and were required to submit their first draft in the format of a Word document. Then, the teacher rated the writings and provided feedback to each student regarding the three dimensions in the rubric. To prepare students for peer assessment activities later, the teacher also selected about 30 representative comments on each class and discussed them with the students in each class in the same week. The example comments covered a wide variety of issues from what the rubric evaluates to suggested problem-solving skills, such as how to utilize online dictionaries and free translation tools to identify the most appropriate words and how to use the corpus to find collocations and confirm pertinent grammatical hypotheses. Then based on teacher feedback, students revised their first drafts, and submitted the revision. To maximize students’ understanding of feedback and promote its take-in, feedback was provided in Chinese or English, as long as the problems could be expounded on clearly. Teacher assessment was done *via* the revision and annotation function of Microsoft Word.

After a 2-week interval, students responded to a second writing task. This time they also took the role of peer raters and were randomly assigned four peer writings on average to assess. Before assessing, a brief in-class training on peer assessment was conducted to explain the requirements as raters. Students reviewed the standards for good writing and were given chances to practice awarding scores. Students then rated peer essays based on the same rubric and provided feedback accordingly. During this process, students were given two different sets of anonymous IDs, one as the rater and one as the essay author. As raters, students were required to provide at least one piece of feedback on each rubric dimension and they were also free to provide feedback in either English or Chinese. There was no restriction on whether they handwrite or type their comments. After they received peer feedback for their essay, students revised the draft, and submitted the revision. To create an incentive (Patchan et al., 2018) and promote the benefits of assessment as learning (Zeng et al., 2018), students were rewarded a participation grade for the accuracy and helpfulness of peer comments.

**Figure 1:**
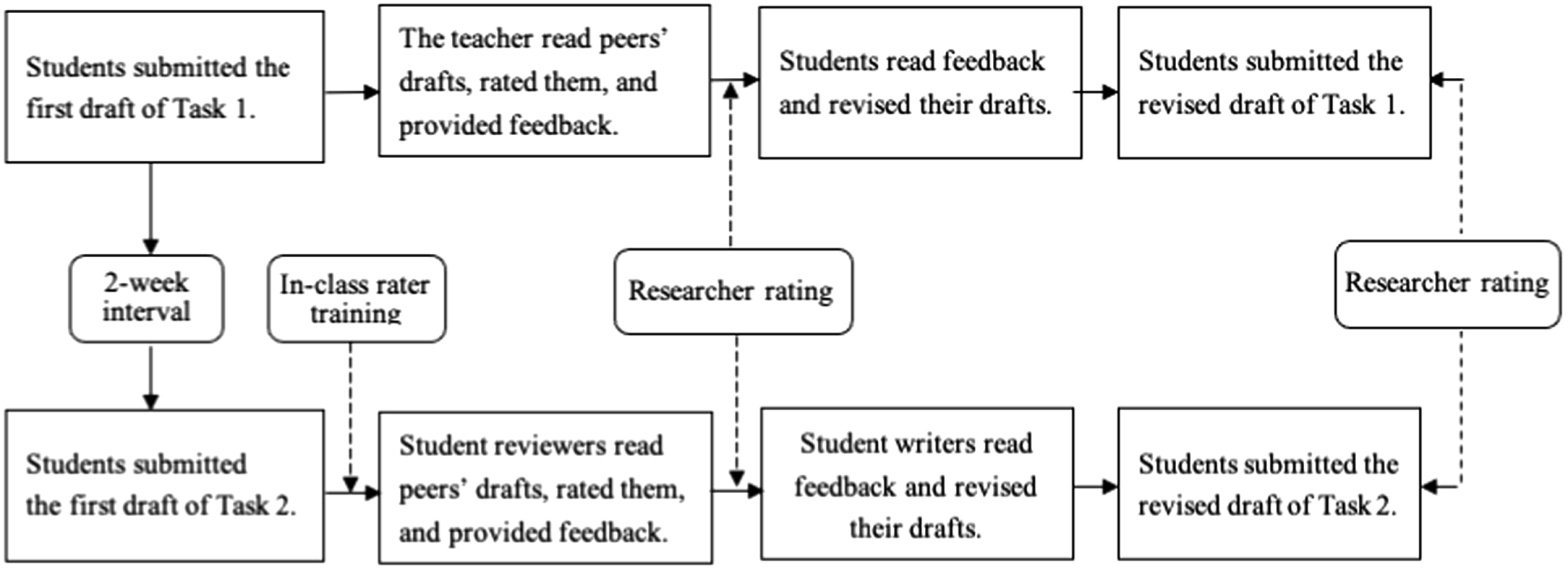
Flowchart of the data collection procedure.

Pre-post comparison on the peer assessment quality was evaluated by the researchers with a partially-crossed design, ensuring that each essay was rated by two raters and each draft received two scores by the same standards. Table 1 presents the rating scheme. In this way, links were established between the rating sessions through common raters. Measurement errors from pertinent facets were thus controlled simultaneously to guarantee valid comparison and data interpretation for this research (Eckes, 2009). The whole data collection procedure is presented in Figure 1.

**Table 1:** Researcher rating of two tasks.

	Task 1		Task 2	
	First draft	Revised draft	First draft	Revised draft
Rater 1	Class A, B, C		Class A, B	Class B, C
Rater 2		Class A, B, C	Class B, C	Class A, C
Rater 3	Samples from Class A, B, C	Samples from Class A, B, C	Class A, C	Class A, B

There was one student who did not submit the essay for Task 1, one student who did not submit the revised draft in Task 2, one student who switched the topic in the revision for Task 2, and one student who did not turn in any work. All of them are males. They were excluded from the final study and analyses below were all done on 90 students in total. We collected and interpreted quantitative data on writing quality to answer the first research question. Then, we interpreted the findings from the qualitative analyses on the feedback to provide crossly-validated evidence for answering the second question.

### Measures

#### Objective measures of text quality

The writing tasks were holistically scored and subjectively rated by teachers or peers as typically done in writing research. However, in addition to that, objective linguistic indicators were also incorporated in this study. The purpose is to supplement the subjective rating with objective criteria and facilitate in-depth diagnoses from more dimensions.

We drew on three natural language processing (NLP) tools, TAACO (Tool for the Automatic Analysis of Cohesion, Crossley et al., 2016, 2019), TAALES (Tool for the Automatic Analysis of Lexical Sophistication, Kyle and Crossley, 2015; Kyle et al., 2018), and TAASSC (Tool for the Automatic Analysis of Syntactic Sophistication and Complexity, Kyle and Crossley, 2018). In general, linguistic features used by researchers in the field fall into three large constructs: lexical, syntactic, and cohesion (Thirakunkovit and Chamcharatsri, 2019). Since NLP tools generally do not measure discourse structures, the quantitative analysis did not include this dimension. We ultimately settled on 22 indices of “logic” and “language quality” where “logic” was measured by 4 cohesion indices and “language quality” by 6 lexical quality indices and 12 syntactic complexity indices.

#### Cohesion

Cohesion refers to “the presence or absence of explicit cues in the text that allow the reader to make connections between the ideas in the text” (Crossley et al., 2016, p. 1128). In this study, we employed the semantic similarity^1^ indices calculated by model word2vec provided by TACCO (version 2.0.4). The four indices quantify similarity between adjacent sentences, between two adjacent sentences, between adjacent paragraphs, and between two adjacent paragraphs, all of which were reported to positively correlate with human judgments of text coherence (Crossley et al., 2019).

#### Lexical quality

Lexical quality falls under the umbrella term of lexical richness, which typically refers to lexical diversity, lexical density, and lexical sophistication. They are frequently used to describe the quality of lexical items (Crossley, 2020). We used TACCO (version 2.0.4) to calculate lexical diversity and density, and TAALES (version 2.2) for lexical sophistication.

Lexical diversity is generally measured by type-token ratio (TTR; e.g., Zhang, 2020), and the index “lemma TTR” in TACCO was used in the current study. Lexical density indicates the proportion of content words and the index of“lexical density (tokens)” in TACCO was selected. Lexical sophistication refers to the learner’s use of sophisticated and advanced words (Kim et al., 2018). According to research, essays with less frequent lexical items are generally thought to be of higher quality and indicate higher writing proficiency (Laufer and Nation, 1995; McNamara et al., 2010a,b; Guo et al., 2013; Kyle and Crossley, 2015; McNamara et al., 2015). Moreover, it has been found that, in addition to word-level frequency, frequency of n-grams and word range are also significant indicators of L2 proficiency (Gries, 2008; Lu, 2010; Crossley et al., 2013). Accordingly, we adopted BNC Written Frequency AW Logarithm, BNC Written Bigram Frequency Logarithm, BNC Written Trigram Frequency Logarithm, and BNC Written Range AW as measures^2^ of lexical sophistication.

#### Syntactic complexity

To examine syntactic complexity, we used the L2 Syntactic Complexity Analyzer (Lu, 2010). It was built into TAASSC (version 1.3.8) and can generate complexity measures of L2 writing automatically for users. Of 14 measures that can be obtained using the analyzer, we included 12 as Yoon and Polio (2017) did in their study because these measures were found to be valid language development indicators (Lu, 2011; Ai and Lu, 2013). All measures and their abbreviations are detailed in the Supplementary Appendix.

#### Feedback coding

**Figure 2:**
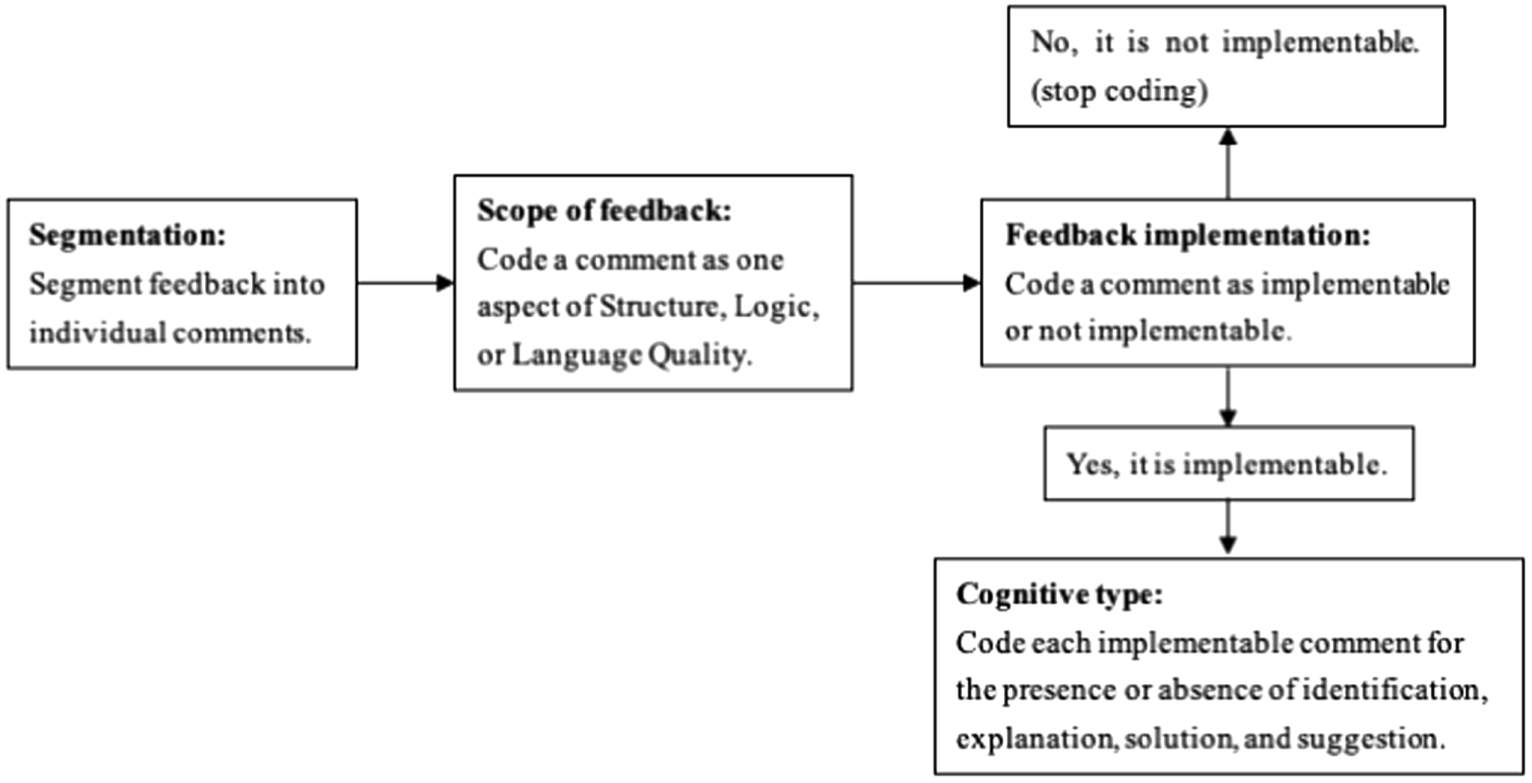
Coding process of feedback (adapted from Wu and Schunn, 2020, 2021).

In order to explore the features of feedback, we coded both teacher and peer feedback, adapting the coding scheme from Wu and Schunn (2020) as presented in Figure 2. Three aspects served as the basis for adaptations. First, we ignored the unimplementable feedback since students could not use them for revision and we focused only on the implementable ones. Second, implementable feedback was only coded for presence or absence of four features: identification, explanation, solution, and suggestion, with affective features *hedges* and *mitigating praise* being neglected since we mainly focus on cognitive aspect in this study. Third, the evaluation rubric was integrated into the *scope* of feedback: the high-level versus low-level classification was replaced with different aspects of Structure, Logic, and Language (Table 2).

**Table 2:** Scope of feedback.

Scope of feedback	Definition	Example
**Structure**		
Holistic structure	Whether the essay has a clear structure, following either a comparison and contrast, cause-effect, or a definition type of essay development	*“However, it seems that the reason of shopping festival’s rise is neglected”*
Presence of topic sentence	Whether there are clear topic sentences for each body paragraph	*“Add clear topic sentences”*
Topic elaboration	Whether topic sentences are well-supported by the details in each paragraph	*“Find some supporting proof instead of making judgement by yourself alone”*
**Logic**		
Sentence-level	Whether transitions between sentences are logical and smooth	*“More transitions between sentences to show logical connection”*
Paragraph-level	Whether transitions between paragraphs are logical and smooth	*“Conclusion is separated from the body part. l do not know where does the viewpoint of ‘whether having siblings is good or bad depends on family education’ come from. It’s a nonsense if it is not coming from the argumentation above”*
**Language quality**		
Accuracy	Whether there are syntactic, morphological, prepositional, and spelling errors	*“‘Why this odd thing happened?’ should be ‘why would this odd thing happened?’”*
Appropriateness	Whether words and phrases are used in the appropriate contexts and whether language use complies with the style of academic writing	*“Expressions are far from native like ‘I’ll say whether having siblings is good or bad depends on family education’”*
Complexity	Whether words, phrases and sentences are complex	*“The expression is simple. For example the expression of ‘get along with”’ has appeared for more than twice in the expository writing”*

As Figure 2 displays, all feedback was cagetogized into two dimensions, *scope* and *cognitive type*. First, feedback was divided into separate idea units. Then the ideas were classified according to the scope structure which was developed in accordance with the rating rubric for the writing tasks in this study (Table 2). Subsequently, we picked out the implementable feedback and coded them for the presence or absence of the four cognitive features in Wu and Schunn (2021): identification, explanation (general) suggestion, and (specific) solution.

There were identical feedback, in terms of both scope and type, received by the same student from different peer raters. In that case, they were counted only once. For instance, if a student received “Your structure is not so clear” and “Structure not clear enough”, the two comments would be coded as one piece of feedback under the scope “holistic clearness”, featured as “identification.”

To ensure reliability of coding, all aspects were meticulously discussed by two researchers in this study, and any inconsistencies were rectified through further discussion with the third researcher. Since the codes are already established, this procedure was time-consuming but not very challenging to achieve consensus. In all, 923 unique idea units were identified from peer feedback and 857 identified from teacher feedback which served as the basis for further analysis.

### Analysis

Firstly, for both Task 1 and Task 2, the rating data were analyzed using Many-Facet Rasch Measurement (MFRM) with the software FACETS (Version 3.82.2; Linacre, 2018). This was done to adjust for measurement error due to rater difference (Aryadoust et al., 2021). Then, to investigate the extent to which peer feedback can supplement teacher feedback in terms of learning outcomes, a series of paired sample *t*-tests were performed for each linguistic index of all writing samples. Meanwhile, feedback was also examined qualitatively to explore the underlying reasons for the discrepancies if there were any.

## Results

### Changes in overall writing quality

We used the fair-averages to compare overall quality of the two drafts for each writing task, which were generated in FACET after controlling for differences among all raters. Students displayed statistically significant score gains in both writing tasks (*t* = 12.23 and 38.79, *p* < 0.001). Thus, Task 2 was not disadvantaged in terms of score improvement despite receiving only peer feedback. In other words, peer feedback can supplement teacher feedback that leads to score gain.

### Differences in revision after peer feedback versus teacher feedback

Tables 3, 4 present the objective cohesion indices before and after teacher and peer feedback, respectively. Writing performance showed significant improvement at both sentence level and paragraph level after teacher feedback based on three out of the four indices. It also improved at both levels after peer feedback, but this was supported only by one indicator per level, both of which seem to measure larger chunks of information.

**Table 3:** Change in cohesion measures after teacher feedback.

Measure	Pre-feedback Mean (SD)	Post-feedback Mean (SD)	*t*	*df*	*p*
**Sentence-level**					
Word2vec similarity (adjacent sentences)	0.832 (0.026)	0.839 (0.027)	3.112	89	0.002**
Word2vec similarity (two adjacent sentences)	0.875 (0.022)	0.880 (0.021)	2.842	89	0.006*
**Paragraph-level**					
Word2vec similarity (adjacent paragraphs)	0.849 (0.068)	0.865 (0.044)	2.190	89	0.031*
Word2vec similarity (two adjacent paragraphs)	0.863 (0.107)	0.876 (0.056)	1.206	89	0.231

*p < 0.05, **p < 0.01, ***p < 0.001.

**Table 4:** Change in cohesion measures after peer feedback.

Measure	Pre-feedback Mean (SD)	Post-feedback Mean (SD)	*t*	*df*	*p*
**Sentence-level**					
Word2vec similarity (adjacent sentences)	0.842 (0.033)	0.845 (0.032)	1.322	89	0.190
Word2vec similarity (two adjacent sentences)	0.880 (0.027)	0.884 (0.026)	2.416	89	0.018*
**Paragraph-level**					
Word2vec similarity (adjacent paragraphs)	0.853 (0.106)	0.872 (0.051)	1.795	89	0.076
Word2vec similarity (two adjacent paragraphs)	0.864 (0.108)	0.889 (0.051)	2.403	89	0.018*

*p < 0.05.

Tables 5, 6 present the statistics for the lexical quality indices. There was no change in lexical diversity or density after either teacher or peer feedback. For lexical sophistication, three out of four indices showed a significant decrease after teacher feedback but only one decreased after peer feedback. In other words, peer feedback is not as influential as teacher feedback in altering lexical sophistication in revisions and revisions tend to show less sophistication.

**Table 5:** Change in lexical quality measures after teacher feedback.

Measure	Pre-feedback mean (SD)	Post-feedback mean (SD)	*t*	*df*	*p*
**Lexical diversity**					
Lemma TTR	0.552 (0.052)	0.548 (0.047)	−0.834	89	0.406
**Lexical density**					
Lexical density (tokens)	0.552 (0.031)	0.555 (0.029)	1.555	89	0.124
**Lexical sophistication**					
BNC written frequency AW logarithm	−0.105 (0.094)	−0.127 (0.081)	−3.632	89	<0.001***
BNC written range AW	75.466 (2.784)	75.004 (2.470)	−2.931	89	0.004**
BNC written bigram frequency logarithm	−1.514 (0.080)	−1.531 (0.070)	−2.589	89	0.011*
BNC written trigram frequency logarithm	−2.245 (0.105)	−2.248 (0.096)	−0.400	89	0.690

*p < 0.05, **p < 0.01, ***p < 0.001.

**Table 6:** Change in lexical quality measures after peer feedback.

Measure	Pre-feedback mean (SD)	Post-feedback mean (SD)	*t*	*df*	*p*
**Lexical diversity**					
Lemma TTR	0.530 (0.048)	0.526 (0.048)	−1.974	89	0.052
**Lexical density**					
Lexical density (tokens)	0.565 (0.035)	0.564 (0.033)	−0.857	89	0.394
**Lexical sophistication**					
BNC written frequency AW logarithm	−0.199 (0.103)	−0.208 (0.106)	−2.113	89	0.037*
BNC written range AW	71.866 (3.955)	71.643 (3.901)	−1.798	89	0.076
BNC written bigram frequency logarithm	−1.553 (0.085)	−1.551 (0.083)	0.382	89	0.703
BNC written trigram frequency logarithm	−2.265 (0.121)	−2.266 (0.112)	−0.023	89	0.982

*p < 0.05.

Tables 7, 8 summarize the changes in syntactic complexity. Nothing changed with statistical significance after peer feedback, but five measures of syntactic complexity involving three dimensions changed significantly after teacher feedback. Teacher feedback increased the length of production unit as measured by MLC (*t* = 1.986, *df* = 89, *p* = 0.050) and also improved coordination as measured by CP/C (*t* = 2.021, *df* = 89, *p* = 0.046). However, the three indices (C/T, DC/C, and DC/T) under Subordination demonstrated a decrease in value instead of an increase after teacher feedback. Neither teacher nor peer feedback had any impact on the three particular structures.

**Table 7:** Change in syntactic complexity measures after teacher feedback.

Measure	Pre-feedback mean (SD)	Post-feedback mean (SD)	*t*	*df*	*p*
**Length of production unit**					
MLS	18.269 (3.466)	17.911 (3.300)	−1.540	89	0.127
MLT	17.459 (3.359)	17.289 (3.197)	−0.730	89	0.468
MLC	10.902 (1.850)	11.150 (1.711)	1.986	89	0.050*
**Subordination**					
C/T	1.622 (0.300)	1.566 (0.275)	−2.237	89	0.028*
DC/C	0.373 (0.094)	0.351 (0.097)	−2.747	89	0.007*
DC/T	0.629 (0.263)	0.573 (0.245)	−2.530	89	0.013*
**Coordination**					
CP/C	0.275 (0.119)	0.292 (0.126)	2.021	89	0.046*
CP/T	0.437 (0.184)	0.447 (0.187)	0.642	89	0.522
T/S	1.052 (0.115)	1.041 (0.100)	−1.143	89	0.256
**Particular structures**					
CN/C	1.354 (0.319)	1.355 (0.283)	0.064	89	0.949
CN/T	2.173 (0.566)	2.109 (0.525)	−1.575	89	0.119
VP/T	2.431 (0.440)	2.383 (0.426)	−1.440	89	0.154

*p < 0.05.

**Table 8:** Change in syntactic complexity measures after peer feedback.

Measure	Pre-feedback mean (SD)	Post-feedback mean (SD)	*t*	*df*	*p*
**Length of production unit**					
MLS	17.892 (3.846)	17.703 (3.390)	−1.018	89	0.312
MLT	16.957 (3.370)	16.740 (2.948)	−1.063	89	0.291
MLC	10.962 (2.244)	10.891 (2.030)	−0.750	89	0.456
**Subordination**					
C/T	1.574 (0.284)	1.558 (0.239)	−0.930	89	0.355
DC/C	0.356 (0.111)	0.351 (0.101)	−0.906	89	0.368
DC/T	0.587 (0.273)	0.567 (0.238)	−1.351	89	0.180
**Coordination**					
CP/C	0.293 (0.139)	0.290 (0.134)	−0.333	89	0.740
CP/T	0.447 (0.196)	0.443 (0.199)	−0.321	89	0.749
T/S	1.057 (0.110)	1.059 (0.098)	0.325	89	0.746
**Particular structures**					
CN/C	1.408 (0.421)	1.397 (0.391)	−0.692	89	0.491
CN/T	2.171 (0.614)	2.146 (0.582)	−0.775	89	0.441
VP/T	2.228 (0.475)	2.203 (0.403)	−0.903	89	0.369

There are too many variables that can influence how feedback is given or taken, such as the proficiency level of raters, familiarity with the essay topics and other considerations at the moment of rating and revising. Thus rather than commenting on the good or badness of the changes in linguistic indices, we simply describe what happened and summarize the general patterns here. In sum, peer feedback seemed to initiate different changes from teacher feedback. While peer feedback seemed to impact the lexical measures, it did not help at the syntactic level. A hypothesis could be that peers do not have the competency to comment on syntactic issues which require higher language proficiency. Teacher feedback did not result in more syntactic complexity and lexical sophistication but these were expected. In fact, feedback analyses showed that the teacher frequently encouraged the students to abort unnecessarily complex words and long sentences for better grammar and organization control. We have also observed that some students seem to hold a misconception that low-frequency words and complex sentence structures equal writing proficiency. Similar guidance had actually been shared with students in the class, however, this might be difficult for students to apply when they gave feedback, which could explain the difference in the linguistic index patterns after teacher and peer feedback.

In all, evidence showed that peers may be able to supplement teacher comments in some aspects which helped raise the scores overall. However, teacher and peer feedback exerted different influences on writing revisions as measured by the linguistic indices. Since implementability of the feedback is an important factor that can influence the revising decisions, we turn to feedback features next to explore their influence on feedback take-in.

### Features of peer feedback versus teacher feedback

In this study, we focused on the cognitive aspect of take-in based on the rubric and implementable feedback framework by Wu and Schunn (2020, 2021). Since the assessment rubric includes Structure, Logic and Language, we categorized the feedback accordingly in these scopes. We then coded all implementable feedback into four types: identification, explanation, solution and suggestion. The latter three types are subsumed under identification, that is, once an issue was identified, the same feedback is further categorized into including an explanation, solution or suggestion. The same identification comment can include both explanation and suggestion, or without any explantion, solution or suggestion, thus the case number in the three sub types did not sum up to the total sum under Identification in the relevant tables below. As a result, independent Chi-square analyses could not be done in some situations where we just presented descriptive statistics instead.

The rightmost column in Table 9 revealed that there is no statistically significant difference between teacher and peers with respect to feedback on Structure (χ^2^ = 1.068, *p* = 0.586).^3^ Both teacher and peers commented on Holistic Structures the most and Presence of Topic Sentence the least. Once the structural issues were identified, the teacher tended to give higher rates of explanation, solution or suggestions than students (52, 10 and 52% vs. 35, 2 and 47%) although the difference in percentages was not significantly different. Relevant distribution of scope elements within each feedback type was not significantly different between the teacher and the peers either. In sum, this group of students were able to evaluate the overall quality of essay structure and they gave similar portions of constructive feedback as the teacher.

**Table 9:** Features of feedback on structure.

		Cognitive type				
		Identification	Explanation	(Specific) solution	(General) suggestion	%
Scope	**Teacher feedback**					
	Holistic structure	33	20	2	18	45%
	Presence of topic sentence	9	2	1	2	12%
	Topic elaboration	31	16	4	18	42%
	Total	73	38	7	38	
	%		52%	10%	52%	
	**Peer feedback**					
	Holistic structure	86	38	1	41	51%
	Presence of topic sentence	15	3	1	6	9%
	Topic elaboration	66	17	1	32	40%
	Total	167	58	3	79	
	%		35%	2%	47%	

Table 10 showed that in terms of Logic, the teacher and peer reviewers identified comparable portions of issues at the paragraph level versus sentence level (χ^2^ = 1.128, *p* = 0.288). Similar to Structure, the teacher was more inclined to offer explanations, solutions or suggestions upon identifying what the problem was. The ratio of these comments were 43, 22, and 23% for the teacher and 32, 6 and 19% for peers. However, the difference did not reach statistical significance within each cognitive type. Again, students seemed to be able to replace the teacher’s role in commenting on logic issues in expository writing.

**Table 10:** Features of feedback on logic.

		Cognitive Type				
		Identification	Explanation	(Specific) solution	(General) suggestion	%
Scope	**Teacher feedback**					
	Paragraph-level	40	18	5	20	29%
	Sentence-level	99	42	26	10	71%
	Total	139	60	31	30	
	%		43%	22%	22%	
	**Peer feedback**					
	Paragraph-level	57	16	0	15	35%
	Sentence-level	105	36	9	16	65%
	Total	162	52	9	31	
	%		32%	6%	19%	

The biggest difference between teacher and peer feedback is on Language Quality. The rightmost column in Table 11 revealed that there was a statistically significant difference between them in terms of their comments on Language Quality (χ^2^ = 95.133, *p* < 0.001). The total amount of issues identified by the teacher far exceeded those by peers (645 vs. 594), and both were much higher compared to those on Structure and Logic. In all, 50% of the teacher feedback focused on Appropriateness, while 51% of peer feedback focused on Accuracy.

**Table 11:** Features of feedback on language quality.

		Cognitive type				
		Identification	Explanation	(Specific) solution	(General) suggestion	%
Scope	**Teacher feedback**					
	Accuracy	160	12	129	2	25%
	Appropriateness	325	96	153	58	50%
	Complexity	160	36	122	19	25%
	Total	645	144	404	79	
	%		22%	63%	12%	
	**Peer feedback**					
	Accuracy	305	41	234	17	51%
	Appropriateness	204	65	72	27	34%
	Complexity	85	14	22	16	14%
	Total	594	120	328	60	
	%		20%	55%	10%	

There was also a difference in the relevant distribution of feedback scopes within Explanation (χ^2^ = 29.580, *p* < 0.001), Solution (χ^2^ = 122.405, *p* < 0.001) and Suggestion (χ^2^ = 21.204, *p* < 0.001). More specifically, both the teacher and peers provided the most explanation on Appropriateness, but the teacher explained Accuracy least while students explained Complexity the least. The teacher provided specific solutions almost equally to all issues identified, but students focused mainly on Accuracy. What’s more, the teacher offered the most suggestions on Appropriateness and the least on Accuracy.

In all, results seemed to imply that students may be able to supplement teacher’s role in identifying the structural and logical issues in expository writing. Although they gave fewer constructive comments compared to the teacher in general, their performance as reviewers was comparable to the teacher’s in providing concrete explanations, solutions and suggestions on both Structure and Logic. This behavior is also reasonable because essay structure and logical relationship between ideas are not as dependent on foreign language proficiency for college-level students as Language Quality is. What they have developed in their first language can be of immediate help in these aspects.

Teacher and student feedback differed most on Language Quality. Some results may be explained by the expertise of teacher over that of students. For example, students may not have the advanced language proficiency to comment on language appropriateness which involves expertise keenness on genre, pragmatics, or cultural awareness. For example, one comment was on the following sentence:

“I can’t feel warmer when she puts up a bright smile and greets me “Welcome home, brother” the moment I arrive home. Honestly speaking, her lovely smile always cures me!”

A student reviewer commented:

“In most cases, “honestly speaking” is used in a relatively negative context. It would be better to use the word “spontaneous” in its place.”

However, “spontaneous” is not pragmatically appropriate, either.

Also, as an experienced instructor, the teacher knew that students were rather weak in using appropriate language in academic writing and was rather sharp on these needs which were a key curriculum goal for the course.

Finally, there were issues that were hard to solve which contributed to the fact that the number of explanations, solutions and suggestions were all lower than the number of issues identified by both the teacher and students. The fact that one student switched to a completely different topic when he was expected to revise the first draft also implied this.

### The connection between feedback and essay quality

The difference in teacher feedback versus peer feedback links back to the essay quality described earlier. In this paper, we present some general patterns which can only be regarded as preliminary.

We noticed that teacher feedback on one linguistic scope such as language appropriateness can exert influence on another, such as language complexity. For instance, replacing “homework” with “assignments” in “there is too much homework for one course” was primarily aimed at improving language appropriateness, however, revision based on this feedback also led to improvement in lexical sophistication since “assignment” is less frequent than “homework”. In another case, the word “affection” was suggested to be replaced with “love” which lowered index value in lexical sophistication. These feedback were both adopted by students and they were examples that could explain the finding why peer feedback was not as influential as teacher feedback in changing lexical sophistication in this study. In addition to this, teacher’s comments on language appropriateness were tailored to individual cases and the detailed explanation can facilitate trust in and take-in of teacher feedback.

Inspection of the feedback also showed that, many peer comments and the teacher comments on language appropriateness were due to the writers’ inaccurate use of advanced words. Consequently, those words were replaced with simpler but more accurate ones in revisions, leading to a decline in lexical sophistication. The same held true for a significant decrease of the three Surbodination indices in syntactic complexity after teacher feedback. For example, comments like “*Stick to only one point within one sentence, and do not make the subject too complicated*” and “*In addition, avoid long sentences. The structure tends to mess up when you use long sentences*” were common. Again, it is apparent from the table that peer reviewers provided a very limited amount of feedback and did not give enough explanations, solutions or suggestions. This led to less take-in and accounted for the fact that no significant changes in syntactic complexity occurred after peer feedback compared to teacher feedback.

To sum up, based on the analyses on linguistic indices and feedback comments, it can be concluded that peer feedback by this group of students was helpful in improving writing performance. It is especially helpful with cohesion and lexical quality of the expository essay, but not so helpful in syntactic complexity. Peer students can identify many issues in writing but they fell short of explanation, solution or suggestion compared to the teacher. This was possibly due to several factors including the shortage of language repertoire to pinpoint the key problem, incompetency to give concrete suggestion or solution, or the play-it-safe mentality in only offering help that they are confident about. However, since we did not interview the students, these remain hypotheses awaiting empirical evidence.

## Discussion

This study examined peer feedback and its take-in by deploying a set of fine-grained measures with three NLP tools. We accounted for different revision outcomes after feedback, and demonstrated how to connect feedback features with the scoring rubric and to evaluate the implementation of comments. Findings indicated that peer feedback can supplement teacher feedback in EFL writing assessment. It led to higher scores overall and altered some linguistic characteristics as measure by the NLP indices. Feedback features by different parties increased our understanding of peer assessment activities and offered some valuable guidance on its implementation in the classroom.

### Implications for peer feedback research

Peer feedback is related to the quality change in student writing. This resonates with previous studies especially when teacher provided training during the peer assessment process (Topping, 1998; van Zundert et al., 2010; Thirakunkovit and Chamcharatsri, 2019; Cui et al., 2021, 2022). However, revision after feedback showed different patterns. Fewer changes in the objective linguistic measures after peer feedback confirmed previous conclusions that teacher feedback was more likely to lead to greater improvements (Yang et al., 2006).

When it comes to feedback type, students often stopped at identifying the language problems, while the teacher tended to give additional explanations as well as solutions and suggestions. This could be due to existent personal and proficiency factors. If English is not the major of the learners and they were caught in all kinds of demanding tasks from numerous courses as in this study, they may not have the motivation to invest much time for the feedback task either. Depending on the specific reason, the solution would vary in order to address these issues. Future studies could explore more on the possible solutions or strategies in order to maximize the value of feedback in the classroom. After all, research should not only address *whether* feedback improves learning, but also *how* it improves learning (Gielen et al., 2010).

Furthermore, it is imperative for feedback studies to draw on more assessment methods. This study adds three NLP tools, showing that they can detect differences between pre-and post-feedback writings that may not be revealed by an overall score. As in Yoon and Polio (2017), not all the indices in this study have been confirmed to be valid developmental measures in previous writing research (which does not imply that they are invalid). However, we are optimistic about using them to track changes in writing ability in future research, especially the measures that demonstrated a significant change in the revisions.

### Implications for classroom teaching and assessment

There is evidence that peer feedback can be regularly exercised in the English classroom writing assessment (Graham and Perin, 2007; Topping, 2009), and this study sheds light on the management of assessment activities in college-level EFL classrooms.

Firstly, it is suggested that peer feedback should be used in combination with teacher feedback to improve pedagogical efficiency. Teachers should pay more attention to those aspects that peers cannot handle. For example, since students commented less on language complexity, this is where teacher feedback should fill in when peer assessment is conducted in the classroom.

More training and instruction should be tuned to complement the limitations of peer feedback as revealed in this study. For example, since students at this level commented less on sentence-level cohesion problems compared with paragraph-level, this means fine discrimination at the sentence level may also be the skill that students are not proficient in. Thus teachers could guide the students to pay more attention to sentence-level cohesion problems and give more examples in class. In addition to that, teachers could guide peer reviewers to explain the problems upon identifying them, since explanatory comments have been found to be positively related to students’ understanding of peer feedback and students’ willingness to respond to it (Gielen et al., 2010; Huisman et al., 2018). If specific instructions are given to students during peer training, it could result in higher improvement in the quality of student writing (Thirakunkovit and Chamcharatsri, 2019).

Results in this study show that not all significant indices were moving in the desired direction. For example, the underlying notion of syntactic complexity is that more complex syntactic structures can act as an indicator of more advanced writing skills. However, three indices (C/T, DC/C, and DC/T) demonstrated a decreasing pattern after teacher feedback, indicating that sentences of the revised drafts were less advanced, such as in Subordination. This actually was not surprising to the teacher., because the teacher had been guiding the students to express themselves as clearly and concisely as possible, and to revise ambiguous, although complicated, sentences. Moreover, sometimes it is not easy to fathom the change in some NLP-based indices. For example, although the indice “lemma TTR” was subsumed under lexical diversity in this study, it is also commonly viewed as an indicator of cohesion, and has been found to be positively related to cohesion measures in previous studies (McCarthy and Jarvis, 2010; Crossley and McNamara, 2014). However, the significant decrease of “lemma TTR” detected after peer feedback contradicted the improvement of cohesion in student revision. Hence, although computer-aided feedback has been proven to increase the total effectiveness of student learning (Lee and Grabowski, 2010), it may not be able to replace human qualitative analysis of writing in the classroom very soon. Research has already shown the limitations of automatic essay scoring and machine learning (Attali and Burstein, 2006; Elliot et al., 2013; Perelman, 2014), this study further shows that rigid linguistic indices may not serve as a quick and adequate fix for issues known and related to human raters (Crossley, 2020).

Finally, similar improvements in scores with different patterns of improvement in language indices demonstrate that assessment in the classroom should be more diversified. Although peer marking is one of the four major interventions for facilitating assessment for learning (AfL; Taras, 2010), it is feedback from peers that can help realize the assessment as learning (AaL) concept in the classrom (Zeng et al., 2018). While human rating remains the golden standard for writing assessment, multiple perspectives of evidence, including the objective indinces can help to assess the progress and change of students’ writing ability in more depth.

## Conclusion

With the help of human rating as well as NLP indices, we draw two main conclusions from this study. Firstly, we confirmed peer feedback as an effective classroom teaching and evaluation method that can assist teacher feedback to improve writing. However, it is limited as measured by a series of objective indices compared to teacher feedback. Secondly, students can identify issues in writing, but provide fewer constructive comments. This may be due to the proficiency of raters which in turn would affect the take-in of the feedback. These are the contributions of this study.

There are some limitations in this study as well, for example, this study provided general patterns mainly with quantitative data and method, we did not interview students nor did we pair up each comment with each revision. Future studies can involve interview data or continue to analyze how each student integrates each feedback in the second draft to reveal individual thinking in addition to the quantitative measures this study has elaborated. This study did not trace the students for longitudinal analyses either. It is possible that language ability develops and changes in an integrated and continuous way. No matter how feedback is provided, as long as there is valuable feedback, students may be able to catch up in every aspect and their writing performance raise to the standards. Another concern that has rarely been discussed in the literature is the redundancy of feedback. In this study, we counted similar remarks as one piece of feedback, however, it is possible that students may implement a feedback more if they notice that it has been mentioned by more raters. Finally, students in this study are highly motivated and advanced learners, generalizability of particular observations in this study may not apply in other contexts. Feasibility and the role of peer feedback may need to be explored case-to-case but we hope the methods we employed can serve as a reasonable example.

## Data availability statement

Rating data and coding results are available upon request from the corresponding author at fchen2020@sjtu.edu.cn.

## Ethics statement

The studies involving human participants were reviewed and approved by the Ethical Review Committee at the School of Foreign Languages, Shanghai Jiao Tong University. The patients/participants provided their written informed consent to participate in this study.

## Author contributions

QS prepared the first drafts of the paper, participated in rating activities and was responsible for NLP indices and feedback coding and analyses. FC designed the study, was responsible for quantitative data analyses and supervised the complete research procedures. SY participated in all coding and rating activities. All authors were involved in the revision processes.

## Conflict of interest

The authors declare that the research was conducted in the absence of any commercial or financial relationships that could be construed as a potential conflict of interest.

## Publisher’s note

All claims expressed in this article are solely those of the authors and do not necessarily represent those of their affiliated organizations, or those of the publisher, the editors and the reviewers. Any product that may be evaluated in this article, or claim that may be made by its manufacturer, is not guaranteed or endorsed by the publisher.

## Footnotes

^1^According to Crossley et al. (2019), how well computational models of semantic memory (Cree and Armstrong, 2012) can highlight underlying semantic relations in texts is a crucial aspect of discourse cohesion in terms of NLP techniques. Also, it exhibited positive relations with measures of cohesion (McNamara et al., 2010a,b).

^2^According to TAALES manual, AW represents “all words,” i.e., both content words and function words. The frequency indices calculate the mean logarithmic frequency for all words to make word frequencies more normally distributed. And all of the four indices are obtained based on ﻿British National Corpus (BNC; BNC Consortium, 2007).

^3^Where there are cells with less than 5 expected counts, we used likelihood ratio test to perform the chi-square analysis.
