# Supplementary material for: The role and features of peer assessment feedback in college English writing
Source: Front Psychol. 2023 Jun 26;13:1070618. doi: 10.3389/fpsyg.2022.1070618 (PMC10332461; doi:10.3389/fpsyg.2022.1070618)
Supplement: Supplementary file 1 [file Table_1.DOCX]

# APPENDIX: Measures of Linguistic Features

| Construct | Measures | Meaning |
| --- | --- | --- |
| *Cohesion* | word2vec similarity (adjacent sentences) | Average word2vec similarity score between all adjacent sentences |
|  | word2vec similarity  (two adjacent sentences) | Average word2vec similarity score between all adjacent sentences with a two-sentence span |
|  | word2vec similarity (adjacent paragraphs) | Average word2vec similarity score between all adjacent paragraphs |
|  | word2vec similarity  (two adjacent paragraphs) | Average word2vec similarity score between all adjacent paragraphs with a two-paragraph span |
| *Lexical quality* |  |  |
| Lexical diversity | lemma TTR | Number of unique lemmas (types) divided by the number of total running lemmas (tokens) |
| Lexical density | lexical density (tokens) | Percentage of text tokens that are content words |
| Lexical sophistication | BNC Written Frequency AW Logarithm | Mean frequency score for all words |
|  | BNC Written Bigram Frequency Logarithm | Mean frequency score for bigrams |
|  | BNC Written Trigram Frequency Logarithm | Mean frequency score for trigrams |
|  | BNC Written Range AW | Mean range (number of documents that a word occurs in) score |
| *Syntactic complexity* | |  |
| ﻿Length of unit | MLS | Mean length of sentence |
|  | MLT | Mean length of T-unit |
|  | MLC | Mean length of clause |
| ﻿Subordination | ﻿C/T | Clauses per T-unit |
|  | ﻿DC/C | Dependent clauses per clause |
|  | ﻿DC/T | Dependent clauses per T-unit |
| ﻿Coordination | ﻿CP/C | Coordinate phrases per clause |
|  | CP/T | Coordinate phrases per T-unit |
|  | T/S | T-units per sentence |
| ﻿Particular structures | ﻿CN/C | Complex nominals per clause |
|  | ﻿CN/T | Complex nominals per T-unit |
|  | ﻿VP/T | Verb phrases per T-unit |
